# Increases in Heroin Overdose Deaths — 28 States, 2010 to 2012

**Published:** 2014-10-03

**Authors:** Rose A. Rudd, Len J. Paulozzi, Michael J. Bauer, Richard W. Burleson, Rick E. Carlson, Dan Dao, James W. Davis, Jennifer Dudek, Beth Ann Eichler, Jessie C. Fernandes, Anna Fondario, Barbara Gabella, Beth Hume, Theron Huntamer, Mbabazi Kariisa, Thomas W. Largo, JoAnne Miles, Ashley Newmyer, Daniela Nitcheva, Beatriz E. Perez, Scott K. Proescholdbell, Jennifer C. Sabel, Jessica Skiba, Svetla Slavova, Kathy Stone, John M. Tharp, Tracy Wendling, Dagan Wright, Anne M. Zehner

**Affiliations:** 1 Division of Unintentional Injury Prevention, National Center for Injury Prevention and Control, CDC; 2 New York State Department of Health; 3 Alabama Department of Public Health; 4 Minnesota Department of Health; 5 Kansas Department of Health and Environment; 6 New Mexico Department of Health; 7 Arizona Department of Health Services; 8 Florida Department of Health; 9 Montana Department of Public Health and Health Services; 10 Utah Department of Health; 11 Colorado Department of Public Health and Environment; 12 Massachusetts Department of Public Health; 13 Nevada Division of Public and Behavioral Health; 14 Ohio Department of Health; 15 Michigan Department of Community Health; 16 New Hampshire Department of Health and Human Services; 17 Nebraska Department of Health and Human Services; 18 South Carolina Department of Health and Environmental Control; 19 Rhode Island Department of Health; 20 North Carolina Department of Health and Human Services; 21 Washington State Department of Health; 22 Indiana State Department of Health; 23 Kentucky Injury Prevention and Research Center; University of Kentucky; 24 Iowa Department of Public Health; 25 Illinois Department of Public Health; 26 Oklahoma State Department of Health; 27 Oregon Health Authority; 28 Virginia Department of Health

Nationally, death rates from prescription opioid pain reliever (OPR) overdoses quadrupled during 1999–2010, whereas rates from heroin overdoses increased by <50%.* Individual states and cities have reported substantial increases in deaths from heroin overdose since 2010. CDC analyzed recent mortality data from 28 states to determine the scope of the heroin overdose death increase and to determine whether increases were associated with changes in OPR overdose death rates since 2010. This report summarizes the results of that analysis, which found that, from 2010 to 2012, the death rate from heroin overdose for the 28 states increased from 1.0 to 2.1 per 100,000, whereas the death rate from OPR overdose declined from 6.0 per 100,000 in 2010 to 5.6 per 100,000 in 2012. Heroin overdose death rates increased significantly for both sexes, all age groups, all census regions, and all racial/ethnic groups other than American Indians/Alaska Natives. OPR overdose mortality declined significantly among males, persons aged <45 years, persons in the South, and non-Hispanic whites. Five states had increases in the OPR death rate, seven states had decreases, and 16 states had no change. Of the 18 states with statistically reliable heroin overdose death rates (i.e., rates based on at least 20 deaths), 15 states reported increases. Decreases in OPR death rates were not associated with increases in heroin death rates. The findings indicate a need for intensified prevention efforts aimed at reducing overdose deaths from all types of opioids while recognizing the demographic differences between the heroin and OPR-using populations. Efforts to prevent expansion of the number of OPR users who might use heroin when it is available should continue.

In February, 2014, CDC invited state health departments to submit data from their mortality files for the period 2008–2012 if they judged those files to be substantially complete and if the causes of death had been coded by the *International Classification of Diseases, 10th Revision*. Participating states had the option of submitting resident deaths or deaths that occurred in the state. States submitted annual counts of deaths with an underlying cause of drug overdose of any intent (codes X40–X44, X60–X64, X85, Y10–Y14). They also submitted counts of subsets of the overdose deaths, those involving heroin (T40.1) and those involving OPR (T40.2–T40.4). States also provided the demographic distributions of these types of overdoses.

CDC calculated annual heroin and OPR death rates per 100,000 using bridged-race population estimates† for each state and for the combined 28 participating states.§ Because examination of state rates revealed pronounced increases in heroin death rates for most states in the study after 2010, CDC calculated changes in rates by demographic characteristics for the period of increasing rates only from 2010 to 2012. The correlation of change in state heroin overdose death rates with change in state OPR overdose death rates was examined both overall and for specific demographic subgroups. Statistical significance of changes in rates was tested using z-tests when rates were based on 100 or more deaths and examination of confidence intervals from gamma distributions when rates were based on fewer than 100 deaths. A weighted Pearson’s correlation coefficient was used to examine the correlation between state level heroin and OPR death rate changes, with weights proportional to the state’s 2012 population. Test results with p≤0.05 were considered statistically significant.

The death rate from heroin overdose doubled in the 28 states from 2010 to 2012, increasing from 1.0 to 2.1 per 100,000 population, reflecting an increase in the number of deaths from 1,779 to 3,635 (Table). Comparing the same years, the death rate from OPR overdose declined 6.6%, from 6.0 to 5.6 per 100,000, a decline from 10,427 to 9,869 deaths. The overall drug overdose death rate increased 4.3%, from 13.0 to 13.6. Heroin death rates increased after 2010 in every subgroup examined. Heroin death rates doubled for males and females, whereas OPR death rates declined 12.4% in males and were unchanged in females. Heroin death rates increased for all age groups, whereas OPR death rates declined for age groups <45 years. OPR death rates increased for persons aged 55–64 years. Heroin death rates doubled in non-Hispanic whites and Hispanic whites, and nearly doubled in blacks. OPR death rates decreased 8% in non-Hispanic whites and remained level in all other races/ethnicities. The Northeast and South had much larger heroin overdose death increases (211.2% and 180.9%, respectively), than the Midwest and West (62.1% and 90.7%, respectively). OPR death rates declined only in the South.

Comparing 2010 to 2012, trends in heroin and OPR overdose death rates varied widely by state. Of the 28 states, five states had increases in OPR death rates, seven states had decreases, and 16 states had no change in the OPR death rate. Of the 18 states with heroin overdose death rates based on at least 20 deaths, none had a decline (Figure 1). Increases in heroin overdose death rates were significantly associated with increases in OPR death rates (r = 0.47, p = 0.05). Similar patterns in the death rates for males and non-Hispanic whites, the two populations with the largest numbers of heroin deaths, also were observed, but the associations were not significant.

In 2012, the age group with the highest heroin overdose death rate was aged 25–34 years, and the age group with the highest OPR overdose death rate was aged 45–54 years. The racial/ethnic population with the highest death rate for both heroin and OPR was non-Hispanic whites (Figure 2). The death rate for heroin among males in 2012 was almost four times that among females, whereas the death rate for OPR among males was 1.4 times that among females.

## Discussion

Combined mortality data from 28 states, encompassing 56% of the U.S. population, indicate an increasing problem with fatal overdoses from heroin from 2010 to 2012. Death rates from OPR declined overall but remained more than twice as high as heroin overdose death rates. Changes in heroin death rates were positively correlated with changes in OPR death rates. Mortality from overdoses of any type of drug rose slightly.

The increase in heroin deaths parallels increases seen in individual states reported previously (1–3). Kentucky reported a 279% increase in heroin deaths from 2010 to 2012 (1). In Ohio, the number of heroin deaths increased approximately 300% from 2007 to 2012, with men aged 25–34 years at highest risk for fatal heroin overdoses (3). Mortality data for the United States show a 45% increase in heroin deaths from 2010 to 2011, the largest annual percentage increase since 1999. The increasing death rate from heroin also is consistent with the 74% increase in the number of current heroin users among persons aged ≥12 years in the United States during 2009–2012 (4). Nationally, OPR death rates from 2010 to 2011 were stable (5.4 per 100,000), although there was a slight increase in the number of OPR deaths.

The rapid rise in heroin overdose deaths follows nearly 2 decades of increasing drug overdose deaths in the United States, primarily driven by OPR drug overdoses (5). The number of persons using OPR nonmedically on a frequent basis also has grown (6). From 2002–2004 to 2008–2010, past year heroin use increased among persons reporting frequent nonmedical use of OPR, from 62.0 to 94.7 per 1,000. Moreover, the only increases in past year heroin use were observed among persons who reported past year nonmedical use of OPR (7). In a sample of heroin users in a treatment program, 75% of those who began opioid abuse after 2000 reported that their first regular opioid was a prescription drug. In contrast, among those who began use in the 1960s, more than 80% indicated that they initiated their abuse with heroin (8). Persons who initiated heroin use after 2000 have reported that heroin often is more readily accessible, less expensive, and offers a more potent high than prescription opioids (8). Although some persons might be discontinuing prescription opioids and initiating heroin use as a replacement, results from this study indicate that recent heroin death rate increases were not significantly associated with decreases in OPR overdose mortality. Numerous risk factors contribute to drug-specific use and overdose death rates (3,8). For example, an increase in overall heroin supply and greater availability of heroin in some parts of the country might contribute to the trend and variation observed in heroin mortality.¶

The findings in this report are subject to at least five limitations. First, death certificates from these states fail to specify the drugs involved in 22% of overdose deaths, so drug-specific overdose rates are underestimated (9). Second, death certificate data might misclassify heroin deaths as OPR deaths if the heroin metabolite morphine is listed on the certificate rather than heroin itself (10). Misclassifications of this type have been demonstrated in several states. However, for this report, this problem is more likely to affect the rates than the percentage changes in those rates. Third, for the 2012 data, six states reported provisional data, and five states reported only deaths that occurred within the state, so the actual rates might vary slightly from those shown. Fourth, the data might reflect fewer than the actual number of deaths for certain racial/ethnic populations because of misclassified, unspecified, or unclassifiable races or ethnicities. In particular, rates by American Indian/Alaska Native populations should be interpreted with caution because of underreporting of these populations. Finally, the data are not necessarily representative of the United States as a whole. Although the distribution of the study population by age, race/ethnicity, and sex closely matched the distribution of the U.S. population, the study population was overrepresented in the Midwest and underrepresented in the West. Because drug overdose death rates vary geographically, trends in this report might differ slightly from overall U.S. trends. Analysis of U.S. trends can be made when mortality files from all 50 states become available.

The findings in this report indicate a growing problem with heroin overdoses superimposed on a continuing problem with OPR overdoses. Increasing use of heroin is especially concerning because it might represent increasing injection drug use. The small decline in OPR overdose mortality is encouraging given its steep increase during 1999–2010 (5), but efforts to address opioid abuse need to continue to further reduce overdose mortality and avoid further enlarging the number of OPR users who might use heroin when it is available. Clinical interventions that might address abuse of both OPR and heroin include screening for substance abuse, urine testing for drug use, and referral to substance abuse treatment. The use of prescription drug monitoring programs can address inappropriate opioid prescribing and further prevent OPR abuse. State policies that increase access to naloxone, a drug that can reverse potentially fatal respiratory depression in persons who have overdosed from either OPRs or heroin, or policies that reduce or eliminate penalties when someone reports an overdose, are potentially useful strategies.** Given the rapid changes in drug overdose epidemiology, timely, drug-specific fatal and nonfatal surveillance data at the local, state, and regional level will be necessary to target prevention efforts.

What is already known on this topic?

A number of jurisdictions in the United States have reported substantial increases in heroin overdose death rates since 2010. Some persons using prescription opioid pain relievers (OPRs) nonmedically have reported switching to or also using heroin.

What is added by this report?

In 28 selected states, representing 56% of the U.S. population, heroin overdose death rates doubled from 2010 to 2012. At the same time, OPR overdose death rates declined 6.6%, and the death rate for drug overdose deaths overall rose 4.3%. Changes in state heroin overdose rates were associated with increases rather than decreases in state OPR overdose death rates.

What are the implications for public health practice?

Timely national, regional, and state surveillance data are necessary to target prevention efforts in the face of rapid changes in drug use patterns that vary across the country. Prevention, treatment, and response strategies that help reduce both heroin and OPR overdose deaths are indicated. Clinical interventions that focus on opioid prescribing, such as screening for substance abuse history and urine testing for drug use, can prevent opioid misuse, particularly for those at high risk for abuse.

## Figures and Tables

**FIGURE 1:**
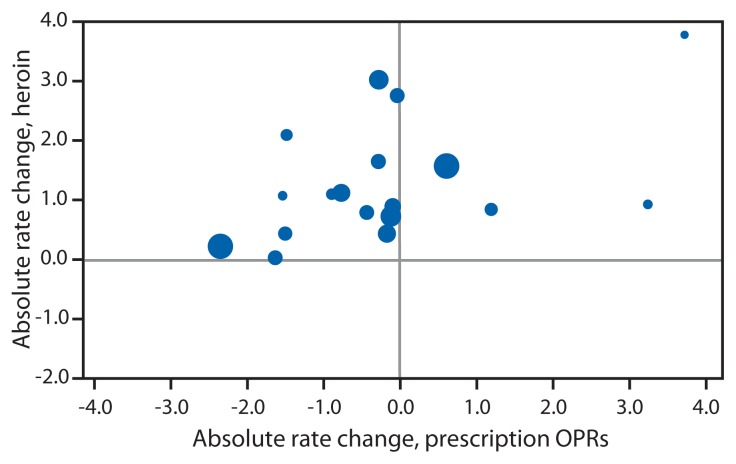
Absolute change in heroin overdose death rates* compared with change in prescription opioid pain reliever (OPR) overdose death rates — 18 states, 2010 to 2012^†^ * Rate change per 100,000 persons (r = 0.47, p = 0.05). Rates based on fewer than 20 deaths in a year are considered unstable and not shown. Marker is proportional in size to the 2012 population of the state it represents. ^†^ Arizona, Colorado, Florida, Illinois, Indiana, Kentucky, Massachusetts, Michigan, Missouri, Nevada, New Mexico, New York, North Carolina, Ohio, Oregon, Utah, Virginia, and Washington.

**FIGURE 2:**
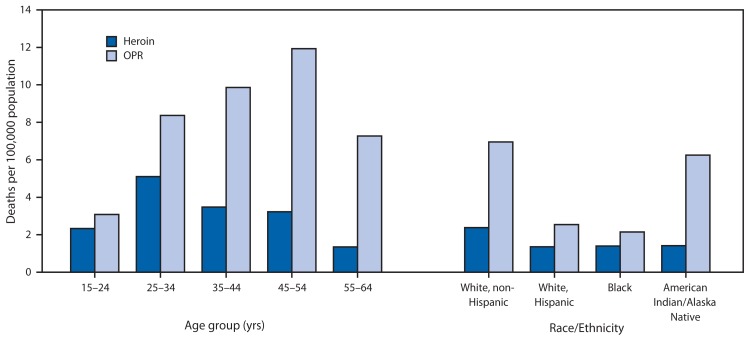
Death rates* from overdoses of heroin or prescription opioid pain relievers (OPRs), by age group and race/ethnicity — 28 states, 2012 * Crude (unadjusted) rate per 100,000 population. Based on bridged-race population estimates for 28 states, available at http://wonder.cdc.gov/bridged-race-v2012.html.

**TABLE:** Annual number of deaths and death rates* from overdoses of heroin or prescription opioid pain relievers (OPRs), by selected characteristics — 28 states, 2008–2012

	Year					Change from 2010 to 2012†	
		
Characteristic	2008	2009	2010	2011	2012	Absolute rate change	% change
**No. of drug overdose deaths**§ **overall**	21,922	22,787	22,472	23,792	23,732	**—**	**—**
Heroin	1,786	2,058	1,779	2,679	3,635	**—**	**—**
OPR	9,480	10,303	10,427	10,393	9,869	**—**	**—**
**Drug overdose death rates overall**	12.9	13.3	13.0	13.7	13.6	**0.6**	**4.3**
Heroin¶	1.0	1.2	1.0	1.5	2.1	**1.0**	**101.7**
OPR**	5.6	6.0	6.0	6.0	5.6	**−0.4**	**−6.6**
**Sex**							
Male							
Heroin	1.7	2.0	1.7	2.5	3.3	**1.7**	**99.0**
OPR	7.0	7.3	7.4	7.1	6.5	**−0.9**	**−12.4**
Female							
Heroin	0.4	0.4	0.4	0.6	0.9	**0.5**	**110.9**
OPR	4.2	4.8	4.7	4.9	4.8	0.1	2.2
**Age group (yrs)**							
15–24							
Heroin	1.2	1.3	1.2	1.9	2.3	**1.1**	**86.3**
OPR	4.1	4.2	4.3	3.8	3.1	**−1.2**	**−28.1**
25–34							
Heroin	2.2	2.7	2.4	3.7	5.1	**2.7**	**109.1**
OPR	8.6	9.2	9.8	9.5	8.4	**−1.4**	**−14.5**
35–44							
Heroin	1.8	2.0	1.8	2.6	3.5	**1.7**	**92.6**
OPR	9.7	10.6	10.5	10.5	9.9	**−0.6**	**−5.9**
45–54							
Heroin	1.8	2.1	1.5	2.2	3.2	**1.8**	**119.6**
OPR	12.0	12.5	12.2	12.3	11.9	**−**0.3	**−**2.5
55–64							
Heroin	0.7	0.8	0.7	1.0	1.3	**0.7**	**102.1**
OPR	5.2	6.4	6.7	6.7	7.3	**0.6**	**8.7**
**Race/Ethnicity** ††							
White, non-Hispanic							
Heroin	1.1	1.3	1.2	1.8	2.4	**1.2**	**101.9**
OPR	6.9	7.4	7.6	7.5	7.0	**−0.6**	**−8.0**
White, Hispanic							
Heroin	1.0	1.0	0.7	1.0	1.4	**0.7**	**102.6**
OPR	2.8	2.5	2.6	2.6	2.5	0.0	**−**0.6
Black							
Heroin	0.8	0.9	0.7	1.0	1.4	**0.7**	**89.3**
OPR	1.8	2.2	2.1	2.0	2.2	0.1	2.7
American Indian/Alaska Native							
Heroin	0.9	1.0	0.9	1.2	1.4	0.6	63.9
OPR	6.2	7.1	6.0	6.2	6.2	0.3	4.5
**U.S. Census region** §§							
Northeast							
Heroin	1.0	1.2	0.9	1.8	2.7	**1.9**	**211.2**
OPR	4.1	4.3	4.3	4.8	4.6	0.3	7.5
Midwest							
Heroin	1.3	1.5	1.6	2.0	2.6	**1.0**	**62.1**
OPR	3.7	4.2	4.3	4.2	4.1	**−**0.2	**−**4.7
West							
Heroin	1.5	1.6	1.2	2.1	2.3	**1.1**	**90.7**
OPR	8.2	8.5	7.9	8.2	7.9	0.1	0.7
South							
Heroin	0.6	0.7	0.4	0.6	1.0	**0.7**	**180.9**
OPR	6.9	7.6	7.9	7.2	6.6	**−1.3**	**−16.3**

* Crude rate per 100,000 population. Based on bridged-race population estimates for 28 states, available at http://wonder.cdc.gov/bridged-race-v2012.html. Because deaths might involve both heroin and OPRs, some deaths are included in both categories.

† Change is in bold if statistically significant (p<0.05). Rate and percentage change might not match calculations based on table data because of rounding.

§ Deaths with underlying causes of unintentional drug poisoning (X40–X44), suicide drug poisoning (X60–X64), homicide drug poisoning (X85), or drug poisoning of undetermined intent (Y10–Y14), as coded in the *International Classification of Diseases, 10th Revision*.

¶ Drug overdose deaths, as defined, that had heroin (T40.1) as a contributing cause.

** Drug overdose deaths, as defined, that had other opioids (T40.2), methadone (T40.3), or other synthetic narcotics (T40.4) as contributing causes.

†† Persons of black and American Indian/Alaska Native race include Hispanic and non-Hispanic ethnicity. Persons of other races/ethnicities or with missing race information on the death certificate are not included.

§§ *Northeast*: Massachusetts, New Hampshire, New York, Rhode Island. *Midwest*: Illinois, Indiana, Iowa, Kansas, Michigan, Minnesota, Missouri, Nebraska, Ohio. *West*: Arizona, Colorado, Montana, Nevada, New Mexico, Oregon, Utah, Washington. *South*: Alabama, Florida, Kentucky, North Carolina, Oklahoma, South Carolina, Virginia.

## Footnotes

* Additional information available at http://wonder.cdc.gov/mcd.html.

† Additional information available at http://wonder.cdc.gov/bridged-race-v2012.html.

§ Alabama, Arizona, Colorado, Florida, Illinois, Indiana, Iowa, Kansas, Kentucky, Massachusetts, Michigan, Minnesota, Missouri, Montana, Nebraska, Nevada, New Hampshire, New Mexico, New York, North Carolina, Ohio, Oklahoma, Oregon, Rhode Island, South Carolina, Utah, Virginia, Washington.

¶ Additional information available at http://www.justice.gov/dea/resource-center/DIR-017-13%20NDTA%20Summary%20final.pdf.

** Additional information available at http://www.cdc.gov/homeandrecreationalsafety/poisoning/laws/index.html.
